# Emergence of Multidrug-Resistant Non-Fermenting Gram-Negative Bacilli in a Tertiary Care Teaching Hospital of Central India: Is Colistin Resistance Still a Distant Threat?

**DOI:** 10.7759/cureus.39243

**Published:** 2023-05-19

**Authors:** Mitisha Soni, Garima Kapoor, Nagaraj Perumal, Deepti Chaurasia

**Affiliations:** 1 Department of Microbiology, Gandhi Medical College, Bhopal, IND; 2 State Virology Laboratory, Department of Microbiology, Gandhi Medical College, Bhopal, IND

**Keywords:** amr, multidrug-resistant acinetobacter, broth microdilution, mcr gene, colistin resistance, mdr non-fermenter

## Abstract

Purpose

Multidrug-resistant (MDR) organisms are being increasingly reported from India. This study aimed to determine the antibiotic susceptibility pattern of non-fermenting Gram-negative bacilli (NF-GNB) isolated from all the clinical samples to estimate the prevalence of MDR MDR NF-GNB and to screen for colistin-resistance genes among all colistin-resistant strains.

Materials and methods

This prospective study conducted from January 2021 to July 2022 at a tertiary care teaching hospital in central India identified MDR NF-GNB from clinical samples using standard procedures and antimicrobial susceptibility testing conducted as per Clinical Laboratory Standards Institute (CLSI) guidelines. Colistin-resistant strains identified by broth microdilution were further subjected to detection of plasmid-mediated colistin-resistant genes (*mcr-1*,* mcr-2*,* mcr-3*) by polymerase chain reaction (PCR).

Results

A total 2,106 NF-GNB were isolated from 21,019 culture positive clinical samples, of which 743 (35%) were MDR. Majority of MDR NF-GNB isolated were from pus (45.50%) followed by blood (20.50%). Out of 743 non-duplicate MDR non-fermenters,the most common were *Pseudomonas aeruginosa *(51.7%)*, Acinetobacter baumannii *(23.4%),and others (24.9%).Around5.2%* Pseudomonas aeruginosa *and 2.3%* Acinetobacter baumannii *were resistant to colistin, and 88.2% were resistant to ceftazidime.* Burkholderia cepacia *complexwas 100% susceptible to minocycline and least susceptible to ceftazidime (28.6%). Out of 11, 10 (90.9%) *Stenotrophomonas maltophilia *were susceptible to colistin and least susceptible to ceftazidime and minocycline (27.3%). All 33 colistin-resistant strains (minimal inhibitory concentration ≥ 4 µg/mL) were found to be negative for *mcr-1*,* mcr-2*, and *mcr-3* genes.

Conclusion

Our study showed a significantly wide variety of NF-GNB, ranging from *Pseudomonas aeruginosa *(51.7%),* Acinetobacter baumannii *(23.4%),to* Acinetobacter haemolyticus *(4.6%), *Pseudomonas putida *(0.9%), *Elizabethkingia meningoseptica *(0.7%), *Pseudomonas luteola *(0.5%), and *Ralstonia pickettii* (0.4%), which have not been commonly reported in literature. Of all the non-fermenters isolated in the present study, 35.28% were MDR, raising the concern for rationalizing antibiotic use and improving infection control measures to avert or slow the emergence of antibiotic resistance.

## Introduction

The upsurge in antimicrobial resistance (AMR) continues to be a global catastrophe [1], and, currently, the world is passing through a consequential yet quiet pandemic of AMR. AMR has been declared by the World Health Organization (WHO) as one of the top 10 global health threats faced by humanity, requiring crucial action to achieve sustainable development goals [2]. AMR led to 1.27 million deaths worldwide in the year 2019 [3] and pushed millions into prolonged illness and hospitalization. If appropriate action against AMR spread is not urgently taken, human casualties could increase to 10 million per year by 2050 [4]. India has been referred to as the AMR capital of the world [5]. The genesis of drug resistance in the Indian context is complex and multifactorial [6].

Globally, more than 71% of all AMR pathogen deaths are caused by *Escherichia coli*, *Staphylococcus aureus*, *Klebsiella pneumoniae*, *Streptococcus pneumoniae*, *Acinetobacter baumannii*, and *Pseudomonas aeruginosa* [2]. Due to a lack of evidence-based data and empirical use of antibiotics in recent years, non-fermenting Gram-negative bacilli (NF-GNB) have become significant pathogens in medical practice, accounting for 12-15 % of all bacterial isolates from clinical samples [7]. They have been linked to infections such as osteomyelitis, septicemia, pneumonia, urinary tract infections, ventilator-associated pneumonia, and surgical site infections [8]. The Centers for Disease Control and Prevention reported 32,600 cases of multidrug-resistant (MDR) *Pseudomonas aeruginosa* infection from hospitalized patients in the United States in 2017, causing 2,700 deaths [9]. Similarly, once *Acinetobacter baumannii* exhibits carbapenem resistance, it has generally acquired resistance to most other antibiotics, leaving fewer therapeutic options [10].

Colistin is used when other antibiotic options are no longer viable for *Acinetobacter baumannii* and *Pseudomonas aeruginosa*. These pathogens are listed as critical priority pathogens for new antibiotics by WHO [11,12]. This illustrates the need to keep colistin as a viable treatment alternative for resistant Gram-negative bacilli. For this, environmental control such as decreasing animal feeds containing colistin or other antibiotics is necessary as expressed by the One Health AMR initiative [13].

Hence, it is crucial to generate local susceptibility data for rapidly evolving and difficult-to-manage MDR pathogens. This study provides the most recent update on colistin resistance and the epidemiology of *mcr-1*, *mcr-2*, and *mcr-3 *genes among MDR NF-GNB in Central India. The detection of plasmid-mediated colistin resistance in *Acinetobacter baumannii* and *Pseudomonas aeruginosa* is important due to the higher chances of spread of colistin resistance in clinical settings [14].

## Materials and methods

**Figure 1:**
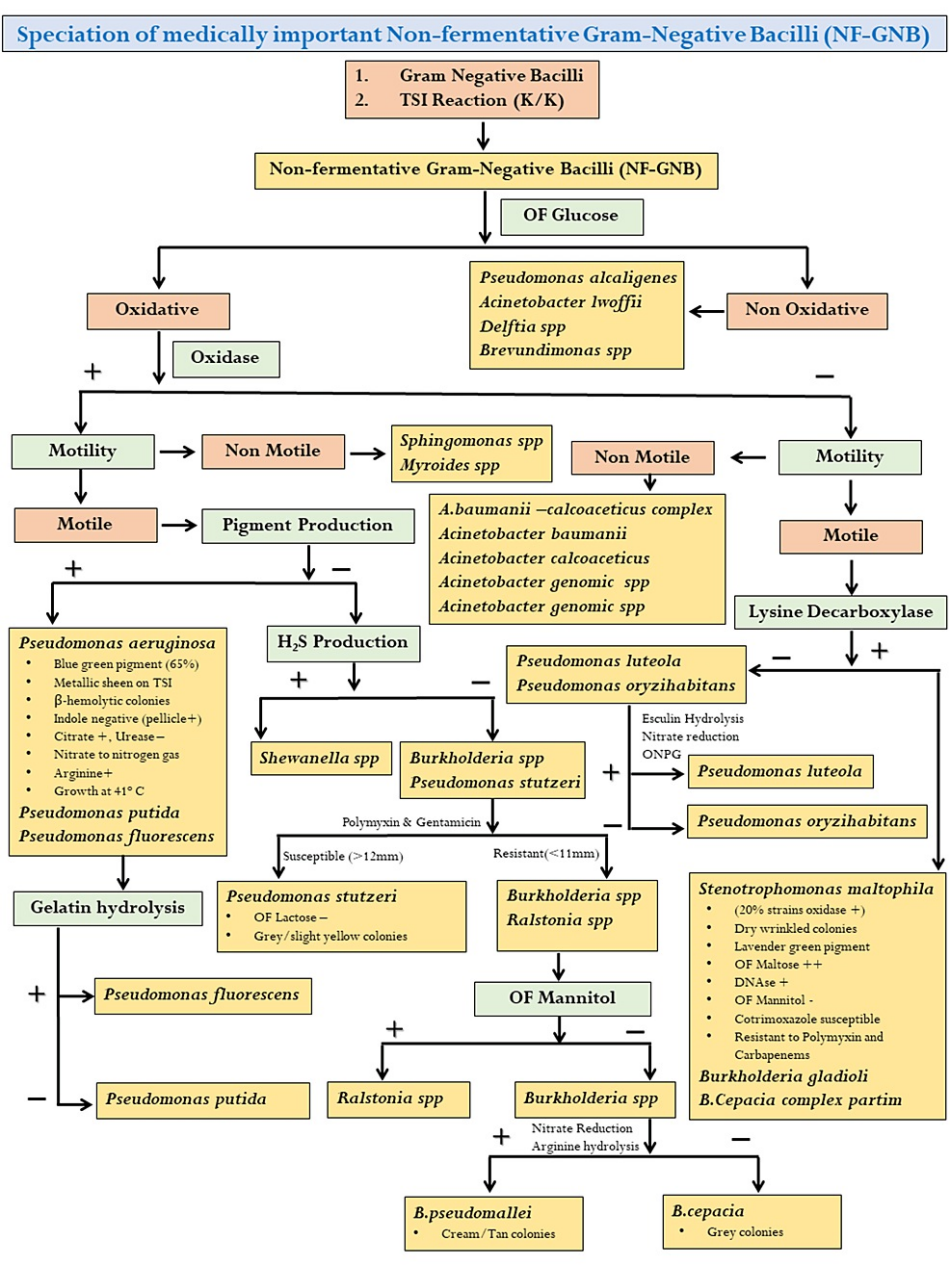
Biochemical test used for the identification of NF-GNB NF-GNB, non-fermenting Gram-negative bacilli

This prospective, observational (non-interventional) study was conducted at the Department of Microbiology of a tertiary care teaching hospital in central India for a duration of 18 months. Patient consent was waived as per institutional ethics committee approval (27088/MC/IEC/2021). The various specimens collected from patients suspected of pyogenic infections were processed for isolation and identification of MDR NF-GNB. All the specimens received in the aerobic section of bacteriology laboratory were inoculated on blood agar, MacConkey agar, and chocolate Agar, and incubated at 37°C in ambient air. Identification of the NF-GNB was done by Gram staining, observing colony morphology, and conventional biochemical methods using the scheme given in Figure 1 [15,16]. A total of 16 isolates, which could not be characterized by conventional biochemical, were identified using GN- 780 card in the VITEK 2 compact system (bioMérieux, Marcy-l'Étoile, France).

The susceptibility of bacterial isolates against different antibiotics was determined by the Kirby-Bauer disk diffusion method, and colistin minimal inhibitory concentration (MIC) was determined by the broth microdilution method (gold standard). Interpretation of antibiotic susceptibility testing was done as per the Clinical and Laboratory Standards Institute (CLSI) guidelines (M100; 32th Edition) [17,18]. Escherichia coli ATCC 25922 and *Pseudomonas aeruginosa* ATCC 27853 were used as the controls for disc diffusion testing. For colistin broth microdilution, *Pseudomonas aeruginosa* ATCC 27853 (MIC: 0.5-4 µg/mL) was used as negative control and an in-house *Pseudomonas aeruginosa* (MIC >16 µg/mL) was used as positive control. The isolates resistant to three or more antibiotic classes were labelled as MDR phenotype [19]. Descriptive analysis of all the observations is performed.

Phenotypically confirmed colistin-resistant strains were further subjected to the detection of plasmid-mediated colistin-resistant genes (*mcr-1*, *mcr-2*, *mcr-3*) by polymerase chain reaction (PCR). DNA extraction was done using the alkaline lysis method. Few colonies were suspended in 100 μL of 25-mM NaOH and kept in a dry bath at 100°C for 10 minutes and then immediately kept in ice bath for 10 minutes. Then, the suspension was neutralized with 8 μL of 1M tris chloride (pH-8). It was then centrifuged, and the supernatant was used as DNA template for the PCR. DNA concentration and quality were determined using a NanoDrop ND‑1000 spectrophotometer (NanoDrop Technologies Inc., Wilmington, DE, USA). Screening by PCR for *mcr-1*, *mcr-2*, and *mcr-3* genes was performed for colistin-resistant isolates by simplex PCR, as described by Al-Kadmy et al. (Table 1) [20]. DNA from a known *mcr-1* positive strain of *Escherichia coli* was used as positive control. Amplification was done using ProFlex PCR System (Applied Biosystems, San Francisco, CA, USA). Electrophoresis of PCR products was carried out at room temperature on horizontal agarose gels (1-2%). Gels containing red stains were run in a gel electrophoresis tank with 1X TAE buffer at 100V for around 30 minutes. After electrophoresis, visualization of the DNA bands was done with the Gel Doc system. The sizes of the PCR products were estimated by comparing their relative mobility with that of 100-bp molecular markers.

**Table 1:** Primers and their sequences used in this study The sequence of primers used in the study were as per Al-Kadmy et al. [20]

Genes	Primer sequences	Product (bp)	Annealing temperature (°C)
mcr-1	F: CACTTATGGCACGGTCTATGA	956 bp	59
	R: CCCAAACCAATGATACGCAT		
mcr-2	F: TGGTACAGCCCCTTTATT	1617 bp	57
	R: GCTTGAGATTGGGTTATGA		
mcr-3	F: TTGGCACTGTATTTTGCATTT	542 bp	50
	R: TAACGAAATTGGCTGGAACA		

## Results

A total 2,106 NF-GNB were isolated from 21,019-culture positive clinical samples accounting for an isolation rate of 10.01%. Of these, 743 were MDR. Most of the NF-GNB isolated were from various medical and surgical wards (56.9%), followed by ICU patients (25.5%) and OPD (outpatient department) patients (17.4%). Majority of MDR NF-GNB isolated were from pus 338 (45.50%), followed by blood 42 (20.50%), urine (12.40%), respiratory samples (11.70%), and sterile fluids (10%). Majority of isolates were from the age group of 46-65 years (49%), and the least were from the age group of 0-1 years (7%).

A total of 743 nonduplicate MDR non-fermenters (*Pseudomonas aeruginosa* [51.7%], *Acinetobacter baumannii* [23.4%], *Acinetobacter lwoffii* [11.3%], *Acinetobacter haemolyticus* [4.6%], *Pseudomonas fluorescens* [2.61%], *Stenotrophomonas maltophilia* [1.5%], *Burkholderia cepacia* [0.9%], *Pseudomonas putida* [0.9%], *Elizabethkingia meningoseptica *[0.7%], *Pseudomonas stutzeri* [0.5%], *Pseudomonas luteola* [0.5%], *Alcaligenes* [0.5%], *Ralstonia pickettii *[0.4%], *Sphingomonas* [0.3%], and *Chryseobacterium indologenes* [0.1%]) were isolated (Table 2).

**Table 2:** Various MDR NFGNB isolated from all clinical samples MDR NF-GNB, multidrug-resistant non-fermenting Gram-negative bacilli

S. no.	MDR isolates	Type of sample					Total
		Urine	Pus	Blood	Respiratory sample	Sterile fluids	
		N (%)					
1	*Acinetobacter haemolyticus*	5 (5.4)	15 (4.5)	7 (4.6)	3 (3.4)	4 (5.4)	34 (4.6)
2	*Acinetobacter baumannii*	20 (21.7)	52 (15.4)	66 (43.8)	9 (10.3)	27 (36.5)	174 (23.4)
3	*Acinetobacter lwoffii*	11 (12.0)	25 (7.4)	22 (14.5)	14 (16.1)	12 (16.2)	84 (11.3)
4	*Alcaligenes*	0 (0.0)	3 (0.6)	0 (0.0)	1 (1.1)	0 (0.0)	4 (0.5)
5	*Burkholderia cepacia complex*	0 (0.0)	2 (0.6)	4 (2.6)	1 (1.1)	0 (0.0)	7 (0.9)
6	*Chryseobacterium-indologenes*	0 (0.0)	0 (0.0)	1 (0.7)	0 (0.0)	0 (0.0)	1 (0.1)
7	*Elizabethkingia meningoseptica*	0(0.0)	1(0.3)	1 (0.7)	1 (1.1)	2 (2.7)	5 (0.7)
8	*Pseudomonas aeruginosa*	56 (60.9)	209 (61.9)	44 (28.9)	53 (60.9)	22 (29.7)	384 (51.7)
9	*Pseudomonas fluorescens*	0 (0.0)	15 (4.5)	1 (0.7)	1(1.1)	2 (2.7)	19 (2.6)
10	*Pseudomonas luteola*	0 (0.0)	2 (0.6)	0 (0.0)	0 (0.0)	2 (2.7)	4 (0.5)
11	*Pseudomonas putida*	0 (0.0)	6 (1.8)	1 (0.7)	0 (0.0)	0 (0.0)	7 (0.9)
12	*Pseudomonas stutzeri*	0 (0.0)	4 (1.2)	0 (0.0)	0 (0.0)	0 (0.0)	4 (0.5)
13	*Raltsonia pickettii*	0 (0.0)	1 (0.3)	1 (0.7)	1 (1.1)	0 (0.0)	3 (0.4)
14	*Sphingomonas*	0 (0.0)	0 (0.0)	0 (0.0)	2 (2.3)	0 (0.0)	2 (0.3)
15	*Stenotrophomonas maltophilia*	0 (0.0)	3 (0.9)	4 (2.6)	1 (1.1)	3 (4.1)	11 (1.5)
	Total	92 (100.0)	338 (100.0)	152 (100.0)	87 (100.0)	74 (100.0)	743 (100.0)

*Pseudomonas aeruginosa *was the most common non-fermenter isolated from pus samples, and *Acinetobacter baumannii* was most frequently isolated from blood cultures. MDR *Pseudomonas aeruginosa* and *Acinetobacter* species were most susceptible to colistin (94.8%, 98%) and least to ceftazidime (16.7%, 4.8 %) (Table 3). *Burkholderia cepacia* complex was 100% susceptible to minocycline and 28.6% susceptible to ceftazidime. Out of 11, 10 (90.9%) *Stenotrophomonas maltophilia* were susceptible to colistin and 27.3% susceptible to ceftazidime and minocycline (27.3%) (Table 4). All 33 colistin-resistant strains (MIC ≥ 4 mcg/mL by broth microdilution) were found to be negative for the most frequently studied [20] plasmid-mediated *mcr-1*, *mcr-2*, and *mcr-3* genes.

**Table 3:** Antibiotic susceptibility profile of multidrug-resistant non-fermenting Gram-negative bacilli

Antimicrobial agents (mcg)	Pseudomonas aeruginosa (%)	Acinetobacter baumannii (%)	Acinetobacter lwoffii (%)	Acinetobacter haemolyticus (%)	Burkholderia cepacia complex (%)	Stenotrophomonas maltophilia (%)
Amikacin (30)	44%	28.7%	15.5%	17.6%	-	
Ceftazidime (30)	16.7%	6.9%	4.8%	2.9%	28.6%	-
Ciprofloxacin (5)	32%	19.0%	21.4%	11.8%	-	-
Gentamicin (10)	43.8%	31.6%	23.8%	20.6%	-	-
Imipenem (10)	33.3%	20.7%	10.7%	14.7%	-	-
Meropenem (10)	56.6%	23.0%	11.9%	8.8%	42.9%	-
Piperacillin-tazobactam (100/10)	34.1%	20.7%	20.2%	14.7%	-	-
Minocycline (30)	-	64.4%	60.7%	64.7%	100%	27.3%
Cotrimoxazole (25)	-	-	-	-	28.6%	36.4%
Ampicillin- sulbactam (10/10)	-	45%	41.6%	41.1%	-	-
Levofloxacin (5)	-	-	-	-	-	27.3%

**Table 4:** Colistin MIC (by broth microdilution method) of all multidrug-resistant non-fermenting Gram-negative bacilli isolated MIC, minimum inhibitory concentration

Isolates	Total number of isolates	Colistin MIC ≤ 2 (intermediate)	Colistin MIC ≥ 4 (resistant)
Acinetobacter haemolyticus	34	32 (94.1%)	2 (5.8%)
Acinetobacter baumannii	174	170 (97.7%)	4 (2.35%)
Acinetobacter lwoffii	84	84 (100%)	-
Alcaligenes faecalis	4	4 (100%)	-
Burkholderia cepacia complex	7	Intrinsically resistant	Intrinsically resistant
Chryseobacterium indologenes	1	-	1 (100%)
Elizabethkingia meningoseptica	5	1 (20%)	4 (80%)
Pseudomonas aeruginosa	384	365 (95%)	19 (5.2%)
Pseudomonas fluorescens	19	19 (100%)	-
Pseudomonas luteola	4	4 (100%)	-
Pseudomonas putida	7	7 (100%)	-
Pseudomonas stutzeri	4	4 (100%)	-
Ralstonia pickettii	3	1 (34%)	2 (66.6%)
Sphingomonas	2	2 (100%)	-
Stenotrophomonas maltophilia	11	10 (90.9%)	1 (10%)

## Discussion

NF-GNB are now emerging as important nosocomial pathogens causing hospital-acquired infections and opportunistic infections. Multidrug resistance has increased among these organisms due to the widespread use of antibiotics [21].

A total of 2,106 NF-GNB were isolated from 21,019 culture-positive clinical samples, accounting for an isolation rate of 10.01%. Out of 2,106 NF-GNB, 743 (35.28%) were MDR, which were similar to the finding by Grewal et al. (11.6%) [8]. The study by Benachinmardi et al. showed an isolation rate of 3.68%. This can be due to difference in the prevalence of resistant bacteria about a decade ago (nine years) and circulation of these bacterial pathogens in different geographical areas [22].

The demographic data from this study showed patients most commonly affected by NF-GNB infections were males between in the age group of 46-65 years (48.7%), which is in concordance with the study conducted by Grewal et al. (40% cases in the age group of 46-65 years) [8]. In this study, most of the MDR NF-GNB were isolated from pus samples (45.5%), followed by blood (20.5%), urine (12.4%), respiratory samples (11.7%), and various body fluids, viz. cerebrospinal fluid and pleural fluid (10%). This is in accordance with a study conducted by Shah and Vaghela, wherein NF-GNB were most commonly isolated from pus samples (73%) [23]. As per literature, carbapenem-resistant *Acinetobacter* is most commonly isolated from wound infections or respiratory specimens. Hence, it is mostly uncertain if it is a colonizer in patients ill for reasons inferable to their underlying condition (e.g., patients requiring mechanical ventilation, patients with extensive burns), or a true pathogen capable of adding to excess mortality, hence the uncertainty about the need for antibiotic therapy [4].

MDR NF-GNB in this study were predominantly from inpatient wards (56.9%), followed by ICU (25.5%) and then OPD (17.4%), similar to the findings reported by Sengupta et al. from the All India Institute of Medical Sciences, Kalyani [24]. Prolonged hospital stay, instrumentation, burns, surgical site infections, prematurity (in case of neonates), diabetes, malignancies, and various underlying ailments predispose these patients to NF-GNB infections [7].

In the present study, *Pseudomonas aeruginosa* was the most common isolate, accounting for 51.7% of all NF-GNB. These results were similar to those reported in previous studies by Jitendranath et al., Juyal et al. [21,25], and Grewal et al. [8], who reported the same species as 57.7%, 38.21%, and 87.96%, respectively. In other studies such as those by Sarkar et al. [26] and Shah and Vaghela [23], *Acinetobacter baumannii *was the most common non-fermenter accounting for 51.3% and 54.0%, respectively, contradictory to our study in which the prevalence of *Acinetobacter baumannii *was 23.4%. The present study also identified *Acinetobacter haemolyticus* (4.6%), *Pseudomonas putida* (0.9%), *Elizabethkingia meningoseptica* (0.7%), *Pseudomonas luteola* (0.5%), and *Ralstonia pickettii* (0.4%), which have not been so frequently reported in the literature.

The MDR *Pseudomonas aeruginos*a isolates showed good susceptibility to colistin (95.3%), followed by meropenem (56.6%), amikacin (44%), gentamicin (43.8%), piperacillin-tazobactam (34.1%), imipenem (33.3%), ciprofloxacin (32%), and ceftazidime (16.7%). This result was in accord with a study conducted by Sarkar et al. (23), which showed high susceptibility to colistin (95%), followed by meropenem (56.4%), amikacin (50.71%), gentamicin (59.2%), piperacillin-tazobactam (30.3%), ciprofloxacin (57.8%), and ceftazidime (23.7%). MDR *Pseudomonas aeruginosa* evolve as a result of an interplay of multiple complex resistance mechanisms, including decreased expression of outer membrane porins (OprD), hyperproduction of AmpC enzymes, upregulation of efflux pumps, carbapenemase production, and mutations in penicillin-binding protein targets [4].

MDR *Acinetobacter baumannii *were highly susceptible to colistin (97.7%) and minocycline (64.4%) but poorly susceptible to ampicillin (16.1%) and ceftazidime (6.9%). In a study on non-fermenters from lower respiratory tract infections, all *Acinetobacter* isolates were MDR and were only susceptible to colistin (100%) [21]. Similar results by Nazir et al. showed that *Acinetobacter baumannii* was highly susceptible to colistin (100%), followed by tigecycline (84%), imipenem (40%), ciprofloxacin (28%), amikacin (26%), and ceftazidime (20%) [27]. The high degree of resistance of *Acinetobacter baumannii *to third-generation cephalosporins could be due to rampant use of this antibiotic in hospital settings, and high susceptibility to colistin might be due to its lesser use as it is an agent of last resort. Carbapenem resistance may be due to OXA24/40-like carbapenemases, OXA-23-like carbapenemases, metallo-β-lactamases, and serine carbapenemases. Sulbactam resistance is mediated via β-lactamase and mutations targeting penicillin-binding proteins. The 16S rRNA methyltransferases or aminoglycoside modifying enzymes lead to aminoglycoside resistance. Quinolone resistance is attributable to mutations in the chromosomally encoded upregulation of efflux pumps [10].

*Burkholderia cepacia* complex was susceptible to minocycline (100%), followed by meropenem (42.9%), cotrimoxazole (28.6%), and ceftazidime (28.6%). *Burkholderia cepacia *complex is intrinsically resistant to polymyxins. Similar results were obtained by Nazir et al.’s study, where *Burkholderia cepacia *complex showed high susceptibility to minocycline (70%) followed by meropenem (67.7%) [27]. However, the studies by Yadav et al. and Sarkar et al. found 100% susceptibility to cotrimoxazole [26,28].

*Stenotrophomonas maltophilia* was susceptible to colistin (90.9%), followed by cotrimoxazole (36.4%), minocycline (27.3%), and levofloxacin (27.3%). The study by Sarkar et al., Nazir et al., and Juyal et al. also showed high susceptibility to cotrimoxazole [25-27]. Antibiotic susceptibility testing for S. maltophilia is tricky. It is intrinsically resistant to majority of the β-lactam antibiotics, including carbapenems. The CLSI has given MIC interpretive criteria for ticarcillin-clavulanate, ceftazidime, cefiderocol, and chloramphenicol, which we could not test due to lack of resources. Also, no CLSI susceptibility criteria are established for polymyxins. Hetero resistance in S. maltophilia is evident by incomplete growth inhibition that often occurs in polymyxin wells. Testing for polymyxin MICs in S. maltophilia is full of challenges, viz. accuracy and reproducibility. Though the use of polymyxins is not suggested by the Infectious Diseases Society of America (IDSA) for the treatment of S. maltophilia infections, we tested colistin broth microdilution using CLSI interpretative criteria for the other two non-fermenters, *Acinetobacter aeruginosa* and *Pseudomonas aeruginosa* [10].

Colistin is used as a last resort drug to treat severe infections caused by MDR non-fermenters. Over the years, the use of colistin has increased by around 10-fold [11]. Due to increased use of colistin, there is rise in resistance. We tested all 743 MDR non-fermenters (except *Burkholderia cepacia* complex, which is known to be intrinsically resistant to colistin) by broth microdilution and found 33 isolates with MIC values ranging from 4 to 16 μg/mL. All the colistin-resistant isolates were evaluated for most frequently studied [11] plasmid-mediated *mcr-1*, *mcr-2*, and *mcr-3* genes. PCR reactions and conditions for each gene are different; therefore, all the three genes were evaluated separately along with DNA ladder and with respective controls. On evaluating, all the three *mc*r genes (*mcr-1*, *mcr-2*, *mcr-3*) were found to be negative on conventional PCR. Further studies will be necessary to determine if the cause of colistin resistance in our strains could be other genes in the *mcr* family (*mcr-4* to *mcr-10​​​​​​)* or might be due to presence of other colistin resistance mechanism such as chromosomal mediated or modification of LPS [29].

Limitations

We could evaluate antibiotic susceptibility for those non-fermenters (*Pseudomonas aeruginosa,* *Acinetobacter *spp., *Stenotrophomonas maltophilia*, and *Burkholderia cepacia* complex) for which disc diffusion interpretive criteria were available in CLSI. Hence, antibiotic susceptibility testing of the rest non-fermenters (6.5 % of total) could not be performed. Colistin broth microdilution was done for all non-fermenters, and interpretative criteria were taken as given in CLSI for *Pseudomonas aeruginosa* and *Acinetobacter *spp. Clinical efficacy trials, correlating patient outcome with definitive antimicrobial treatment instituted, would further generate strengthened data for patient welfare. This is a single-center study; hence, the findings are impacted by the local epidemiology factors and therefore should be repeated at other institutions. Also, further molecular evaluation of the cause of colistin resistance could have been performed.

## Conclusions

With the sharp rise in MDR Gram-negative bacteria, especially non-fermenters, it is essential for clinicians of each region to remain updated with the latest prevalence and antimicrobial susceptibility pattern of the circulating pathogens. A high prevalence (35.3%) of MDR *Pseudomonas aeruginosa *and MDR *Acinetobacter baumannii *in the present study raises the concern of rapidly emerging antibiotic resistance in this group of bacteria in our region. Additionally, these bacteria have the capability to survive in hospital environment, and thus improved infection‑control measures and antibiotic stewardship are essential to prevent or slow their emergence and spread.

Polymyxins are increasingly used as the last viable therapeutic option. In cases of multidrug resistance, colistin can be used in synergy with other antimicrobials. The screening for plasmid-mediated colistin resistance in *Acinetobacter baumannii *and *Pseudomonas aeruginosa* is important. Knowing the molecular mechanism of drug resistance transmission will guide in controlling the spread of colistin resistance in clinical settings. It is important to analyze and develop guidelines for use of colistin in order to ensure that this treatment option remains viable.
